# Live-cell imaging of macrophage phagocytosis of asbestos fibers under fluorescence microscopy

**DOI:** 10.1186/s41021-019-0129-4

**Published:** 2019-06-05

**Authors:** Takenori Ishida, Nobutoshi Fujihara, Tomoki Nishimura, Hisakage Funabashi, Ryuichi Hirota, Takeshi Ikeda, Akio Kuroda

**Affiliations:** Department of Molecular Biotechnology, Graduate School of Advanced Sciences of Matter, Hiroshima University, 1-3-1 Kagamiyama, Higashi Hiroshima, Hiroshima, 739-8530 Japan

**Keywords:** Asbestos, Frustrated phagocytosis, Multinucleation

## Abstract

**Background:**

Frustrated phagocytosis occurs when an asbestos fiber > 10 μm in length is engulfed imperfectly by a macrophage, and it is believed to be associated with chromosomal instability. Few studies have focused on dynamic cellular imaging to assess the toxicity of hazardous inorganic materials such as asbestos. One reason for this is the relative lack of fluorescent probes available to facilitate experimental visualization of inorganic materials. We recently developed asbestos-specific fluorescent probes based on asbestos-binding proteins, and achieved efficient fluorescent labeling of asbestos.

**Results:**

Live-cell imaging with fluorescent asbestos probes was successfully utilized to dynamically analyze asbestos phagocytosis. The fluorescently labeled asbestos fibers were phagocytosed by RAW 264.7 macrophages. Internalized fibers of < 5 μm in length were visualized clearly via overlaid phase contrast and fluorescence microscopy images, but they were not clearly depicted using phase contrast images alone. Approximately 60% of the cells had phagocytosed asbestos fibers after 2 h, but over 96% of cells remained alive even 24 h after the addition of asbestos fibers. Immediate cell death was only observed when an asbestos fiber was physically pulled from a cell by an external force. Notably, at 24 h after the addition of asbestos fibers an approximately 4-fold increase in the number of binucleated cells was observed. Monitoring of individual cell divisions of cells that had phagocytosed asbestos suggested that binucleated cells were formed via the inhibition of cell separation, by asbestos fibers of > 10 μm in length that were localized in the proximity of the intercellular bridge.

**Conclusions:**

Fluorescently labeled asbestos facilitated visualization of the dynamic biological processes that occur during and after the internalization of asbestos fibers, and indicated that (i) frustrated phagocytosis itself does not lead to immediate cell death unless the asbestos fiber is physically pulled from the cell by an external force, and (ii) macrophages that have phagocytosed asbestos can divide but sometimes the resulting daughter cells fuse, leading to the formation of a binucleated cell. This fusion only seemed to occur when a comparatively long asbestos fiber (> 10 μm) was shared by two daughter cells.

**Electronic supplementary material:**

The online version of this article (10.1186/s41021-019-0129-4) contains supplementary material, which is available to authorized users.

## Introduction

Asbestos is a fibrous silicate mineral that has been widely used in construction materials because of its useful properties, such as high ultimate tensile strength, low thermal conduction, and relative resistance to chemical attack [1, 2]. Inhalation of asbestos fibers was found to damage the lungs however, resulting in serious health problems such as pleural mesothelioma and lung cancers [3–6]. Although the use of asbestos has now been prohibited in most developed countries, asbestos remains in old buildings. Renovation or demolition of these buildings can result in the release of airborne asbestos fibers into the environment. Asbestos is still common in most of the world, and the incidence of asbestos-linked cancers continues to rise [5, 7].

Inhaled asbestos fibers reach the pulmonary alveoli and cause an inflammatory response [8, 9]. Alveolar macrophages phagocytose particles such as dust and microorganisms, and remove them from the respiratory surfaces. They also try to remove asbestos fibers via phagocytosis [10, 11]. Relatively short fibers are apparently fully enclosed in the phagosomes and cleared, and it has been demonstrated that fibers < 5 μm in length are not retained in the lungs and do not cause chronic inflammation [12]. In contrast, longer fibers evidently can not be fully engulfed by macrophages—resulting in a process called frustrated phagocytosis—and can remain inside the lung for a long time [13, 14]. Phagocytosed long asbestos fibers activate the NLRP3 inflammasome, leading to maturation of interleukin (IL)-1ß, which triggers inflammation [15]. The longer fibers that are not cleared lead to chronic inflammation, which exerts pleiotropic effects in the development of cancer.

With regard to asbestos toxicity, several aspects pertaining to the survival and division of macrophages that have phagocytosed asbestos remain unclear. It has been suggested that frustrated phagocytosis results in failure of membrane closure, resulting in the leakage of cell contents [14]. The leakage of cell contents may lead to immediate cell death. However, whether frustrated phagocytosis leads to immediate cell death remains to be elucidated. While investigating cell division, Jensen et al. [16] demonstrated that long fibers phagocytosed by monkey epithelial cells sterically blocked cytokinesis, sometimes resulting in the formation of a binucleated cell. Asbestos-induced polyploidy and chromosomal instability may facilitate tumor initiation and/or speed up tumor development [17]. Macrophage-derived multinucleated giant cells (MGCs) were observed in asbestos-exposed mouse lungs [18]. Factors inducing MGC formation are not well defined [19], but IL-4, a T_H_2 cytokine associated with macrophage activation, induces fusion [20, 21]. The mechanisms related to asbestos-derived MGC formation remain to be elucidated.

The dynamic biological processes that occur during and after the internalization of asbestos fibers have not been clarified. One of the reasons is the relative lack of development of dynamic analytical methods such as live cell imaging for the study of asbestos in this context. Cellular internalization of asbestos fibers has been analyzed in numerous studies using transmission electron microscopy or combinations of atomic force microscopy and soft x-ray microscopy [16, 22–24]. Although these methods provide information in finer detail than light microscopy, they are not suitable for analyzing dynamic biological processes during asbestos phagocytosis in conjunction with biomolecule co-localization with internalized asbestos fibers. A typical strategy for conducting these types of analyses is live-cell imaging incorporating fluorescence microscopy (FM). We recently developed fluorescent asbestos probes based on asbestos-binding proteins, and have achieved efficient fluorescent labeling of asbestos [25]. The thinnest fibers of chrysotile asbestos visualized under FM in that study were approximately 30–35 nm in diameter as estimated via scanning electron microscopy, which is similar to the reported diameters of single chrysotile fibrils [25]. In the present study, to analyze asbestos phagocytosis and its subsequent biological processes dynamically, we utilized fluorescently labeled asbestos and live-cell imaging in conjunction with confocal laser scanning microscopy (CLSM).

## Materials and methods

### Materials

Amosite (JAWE231) and crocidolite (JWE331) asbestos were obtained from the Japan Association for Working Environment Measurement (Tokyo, Japan). Unless otherwise noted, amosite asbestos was used in the experiments. Dulbecco’s modified Eagle’s medium (DMEM) with high glucose and HEPES and without phenol red, fluorescently labeled streptavidin (streptavidin-Cy3, streptavidin-DyLight488), and actin fluorescent stain reagent (ActinGreen™ 488 Ready Probes Regent) were purchased from Thermo Fisher Scientific (Hanover Park, IL, USA). Fluorescently labeled streptavidin (streptavidin-CF555) was purchased from Biotium Inc. (Hayward, CA, USA). All other reagents were purchased from Wako Chemicals (Tokyo, Japan) or Sigma (St. Louis, MO, USA).

### Cell culture

RAW 264.7 cells were purchased from DS Pharma Biomedical Co., Ltd. (catalogue number EC91062702-F0; Osaka, Japan). Cells were cultured in DMEM containing 10% heat-inactivated fetal bovine serum (HyClone FBS; GE Healthcare Life Sciences, Logan, UT, USA), 1% non-essential amino acids solution (MEM, Nakarai Tesque, Kyoto, Japan), 100 units/mL penicillin, and 0.1 mg/mL streptomycin at 37 °C and 5% CO_2_ in a humidified incubator.

### Preparation of fluorescent probes and fluorescent labeling of asbestos fibers

The preparation of fluorescently labeled streptavidin tetramer complexes was conducted as previously described [26]. Briefly, 1 μM streptavidin-Cy3, streptavidin-CF555, or streptavidin-DyLight488 was mixed with 20 μM biotinylated H-NS_60–90_ fragment (Siliconbio Inc., Hiroshima, Japan) in 0.1 M Tris-HCl buffer (pH 8.0) and incubated for 1 h. Fluorescently labeled streptavidin tetramer was diluted to 20 nM in 0.5 mL of PBS, mixed with 0.1 mg of asbestos, and incubated for 1 h at room temperature with rotary mixing. The fluorescently labeled asbestos was precipitated via centrifugation at 12,000 *g* for 3 min. The precipitated fibers were washed three times with 0.5 mL of PBS, precipitated via centrifugation at 12,000 *g* for 3 min, then resuspended in 0.1 mL of PBS. The zeta-potentials of unlabeled and fluorescently labeled asbestos fibers were measured using a zeta potential and particle size analyzer (ELS-Z, Otsuka Electronics Co., Ltd., Japan).

### Continuous monitoring of asbestos fiber phagocytosis by RAW 264.7 cells

RAW 264.7 cells were plated in glass-bottomed culture dishes (35/10 mm; Greiner Bio-One, Frickenhausen, Germany) at a density of 2 × 10^5^ per dish and cultured for 2 days at 37 °C and 5% CO_2_ in a humidified incubator. The medium was then replaced with 2 mL of fresh medium and the cells were incubated in the absence of asbestos fibers or in the presence of 50 μg/mL of Cy3-labeled asbestos fibers. During live imaging, the cells were incubated in a stage-top incubator (TOKAI HIT, Shizuoka, Japan) at 37 °C and 5% CO_2_. Images were then obtained continuously every 5 min for 15 h using an IX71 fluorescence microscope (Olympus, Tokyo, Japan) equipped with an EM-CCD camera (C9100–13; Hamamatsu Photonics K.K., Shizuoka, Japan).

### Determination of cell survival

RAW 264.7 cells were plated at a density of 2 × 10^4^ cells per well in a 4-well glass-bottomed dish (Matsunami Glass Inc. Ltd., Osaka, Japan) and cultured for 1 day in a humidified incubator at 37 °C and 5% CO_2_. The medium was then replaced with 0.2 mL of fresh medium and the cells were incubated in the absence of asbestos fibers or in the presence of 25 μg/mL of unlabeled or DyLight488-labeled asbestos fibers. The LIVE/DEAD™ viability kit for microscopy and quantitative assays (Thermo Fisher Scientific, Hanover Park, IL, USA) was used to determine cell survival via propidium iodide uptake, by counting unstained (viable) and stained (non-viable) cells under a fluorescence microscope (BIOREVO BZ-9000, Keyence, Osaka, Japan).

### Observation of asbestos inside RAW 264.7 cells via CLSM

RAW 264.7 cells were plated at a density of 5 × 10^4^ cells per well in a 4-well glass-bottomed dish (Matsunami Glass Inc. Ltd.) and cultured for 2 days in a humidified incubator at 37 °C and 5% CO_2_. The medium was then replaced with 0.2 mL of fresh medium containing 25 μg/mL of Cy3 or CF555- labeled asbestos, and the cells were incubated at 37 °C and 5% CO_2_. After 2 h the cells were washed with PBS then fixed with 4% paraformaldehyde in PBS for 10 min. They were then washed twice with PBS, followed by 0.5% Triton X-100 treatment for 10 min at 37 °C. Lastly, the cells were stained with ActinGreen™ 488 and DAPI (Sigma) in accordance with the manufacturer’s protocol. Images were obtained using a confocal laser scanning microscope (Olympus FV1000, Olympus Optical Co. Ltd., Tokyo, Japan) in channel mode with 405, 473, and 559 nm excitations. The resulting fluorescence emissions were acquired using 425–475-nm (for DAPI), 515–565-nm (for ActinGreen™ 488), and 570–625-nm (for Cy3 and CF555) band-pass filters. Detailed 3D images were constructed using Olympus FV10-ASW software. The numbers and lengths of internalized asbestos fibers in cells were measured.

### Counting multinucleated cells

RAW 264.7 cells were plated at a density of 2 × 10^4^ cells per well in a 4-well glass-bottomed dish (Matsunami Glass Inc. Ltd.) and cultured for 1 day in a humidified incubator at 37 °C and 5% CO_2_. The medium was then replaced with 0.2 mL fresh medium and the cells were incubated in the absence of asbestos fibers or in the presence of 25 μg/mL of unlabeled or Cy3-labeled asbestos fibers. The doubling time of RAW 264.7 cells is reported 11–12 h [27]. At 6, 12, and 24-h time-points cells were fixed, stained with DAPI and ActinGreen™ 488, and examined using a fluorescence microscope (BIOREVO BZ-9000). The numbers of cells with one nucleus and two nuclei (binucleated) were recorded, and as were the numbers of multinucleated (i.e., > two nuclei) cells.

### Statistical analysis

Statistical analysis was performed using the two-tailed unpaired Student’s *t*-test, and *p* < 0.05 was considered statistically significant.

## Results and discussion

### Fluorescent labeling of asbestos fibers

**Fig. 1:**
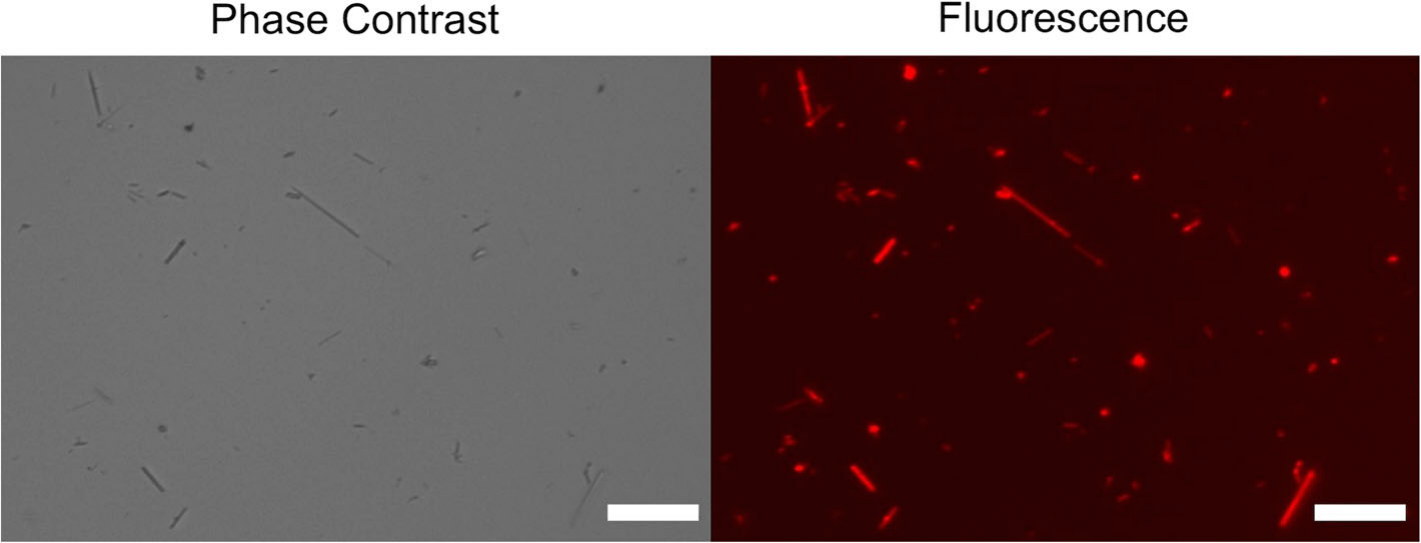
Phase contrast and fluorescence images of asbestos fibers. Asbestos was stained with a fluorescent probe. Phase contrast and fluorescence images of asbestos fibers were acquired in the same field of view. Scale bar = 20 μm

*Escherichia coli* H-NS protein has the ability to bind to all types of amphibole asbestos [26]. A 31-amino acid peptide responsible for the affinity of H-NS protein to asbestos was displayed on a commercially available fluorescently labeled streptavidin tetramer (asbestos-fluorescent probe). The asbestos-fluorescent probe can bind to asbestos with a dissociation constant of 1.0 nM [26]. A mixture of 1 μg of asbestos-fluorescent probe and 100 μg of asbestos (1.4 × 10^7^ fibers) was observed under FM (Fig. 1). Under these conditions approximately 4200 probe molecules bound to one asbestos fiber [26]. The length distribution of the fibers was determined to be 3.6 ± 0.4 μm using CLSM images (see below).

In a two-tailed unpaired Student’s *t*-test including data from *n* = 4 preparations, there was no significant difference between the mean zeta potential of asbestos (− 25.47 ± 0.83 mV) and that of fluorescently labeled asbestos (− 24.48 ± 0.55 mV). As described below, cell survival was not changed when asbestos and fluorescently labeled asbestos fibers were phagocytosed. Furthermore, the frequency of binucleated cell formation in the presence of fluorescently labeled asbestos fibers was almost same as that in the presence of unlabeled asbestos fibers. These results suggested that fluorescent labeling of asbestos had little or no effect on cellular interaction with the fibers.

### Macrophage phagocytosis of asbestos fibers

**Fig. 2:**
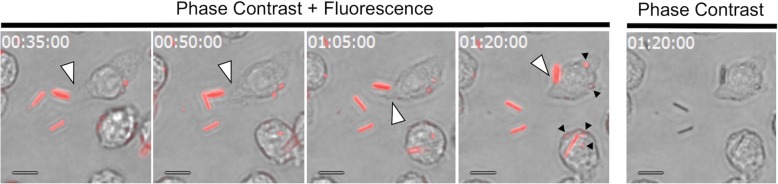
Live-cell imaging of asbestos phagocytosis. Phagocytosis of fluorescently labeled asbestos fibers by RAW 264.7 cells (white arrowhead) was depicted using phase contrast images overlaid with fluorescence microscopy images. Small black arrowheads indicate short fibers. The images are from the field of view shown in Additional file 1, and the numbers in the in the upper left corner indicate time (h: min: sec). Scale bar = 10 μm

RAW 264.7 macrophages were used to investigate the biological effects of asbestos fibers in vitro [28, 29]. Fluorescently labeled asbestos fibers were phagocytosed by RAW 264.7 macrophages (Fig. 2, white arrowheads, Additional file 1). The phagocytosis of short fibers (Fig. 2, small black arrowheads) was clearly depicted in phase contrast images overlaid with fluorescence microscopy images, but not in phase contrast images alone. This was due to the presence of intracellular organelles with similar dimensions.

**Fig. 3:**
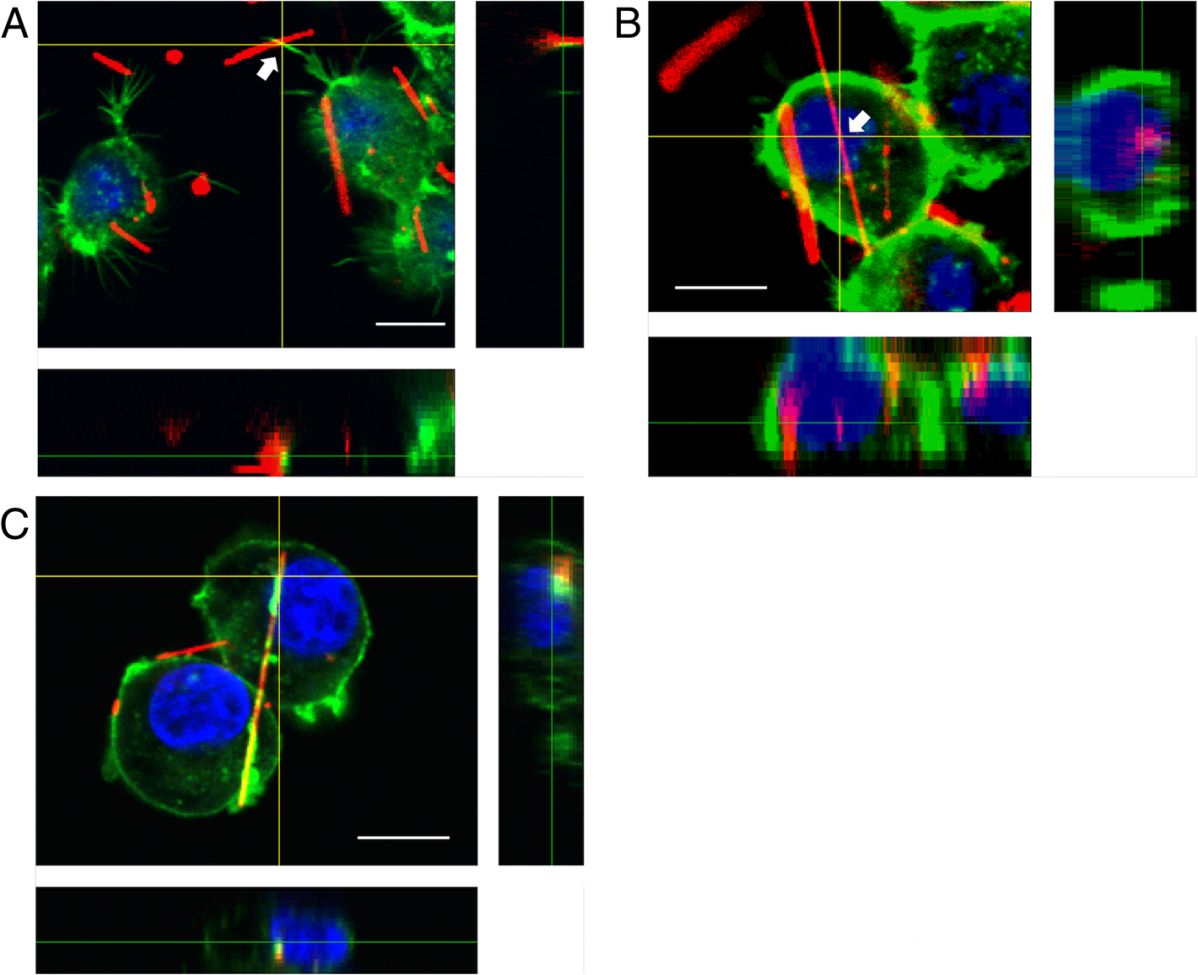
Confocal laser scanning microscopy images of phagocytosis of fluorescently labeled asbestos fibers by RAW 264.7 cells. RAW 264.7 cells were incubated with fluorescently labeled crocidolite (red) (**a**, **b**) and amosite (red) (**c**) asbestos for 2 h. The actin cytoskeleton and nuclei of the cells were respectively stained with ActinGreen™ 488 (green) and DAPI (blue). Z-axis images at vertical and horizontal yellow lines were extracted from 3D images, and respectively indicate right and bottom positions. Scale bar = 10 μm

Macrophages can make contact with suspicious particles via long tentacle-like protrusions called filopodia, pull them towards their cell bodies, then internalize and destroy them. The internal scaffolds of filopodia are long, and their dynamic filaments of actin can be visualized via ActinGreen™ 488. Capturing multiple 2D images at different depths using CLSM enables the reconstruction of 3D structures within a cell. Figure 3a (arrow) and its z-axis images show an asbestos fiber (red) captured by filopodia (green) prior to phagocytosis.

**Fig. 4:**
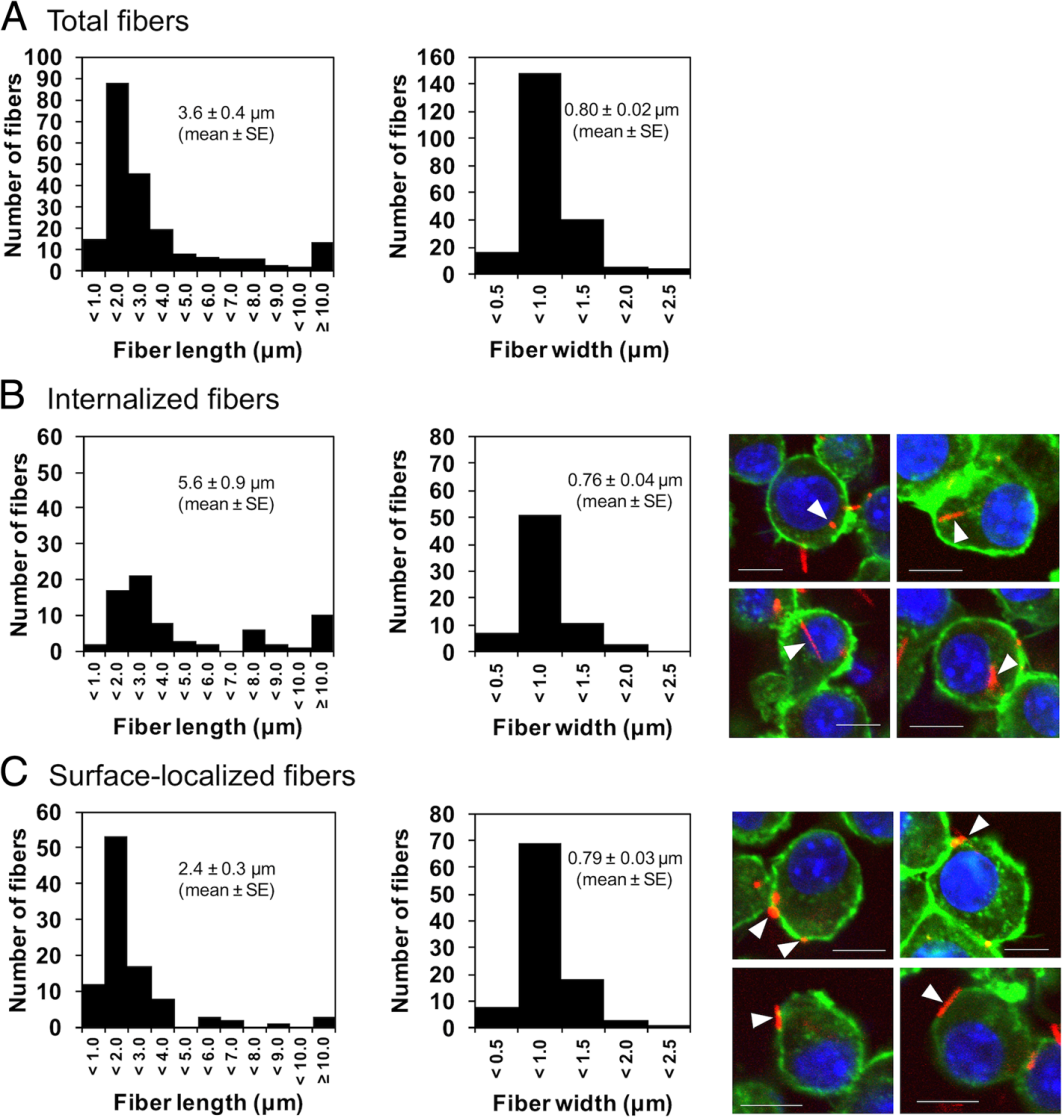
Length and width distributions of asbestos fibers. Graphs show the length and width distributions of the total fiber preparation (**a**), internalized fibers (**b**), and surface-localized fibers (**c**). Fiber lengths and widths were measured using confocal laser scanning microscopy images. Typical images are shown on the right side (arrowheads indicate fluorescently labeled fibers). Scale bar = 10 μm

A reconstructed CLSM image depicting the internalization of crocidolite (Fig. 3b) and amosite (Fig. 3c) fibers longer than 10 μm (frustrated phagocytosis) was generated. Z-axis images indicated that a long asbestos fiber was partially internalized, but not localized on the cell surface (Fig. 3b, arrow). We determined the length distribution of total fibers the cells were exposed to (Fig. 4a). The mean length of internalized fibers (5.6 ± 0.9 μm) (Fig. 4b) was longer than that of the total fibers (3.6 ± 0.4 μm) (Fig. 4a). Interestingly, we found that the mean length of surface-localized fibers (2.4 ± 0.3 μm) (Fig. 4c) was approximately half that of internalized fibers. Although the cells phagocytosed both short and long fibers, the intracellular localization of asbestos depended on fiber length. On the other hand, the width distribution of the total, internalized, and surface-localized fibers were very similar (Fig. 4), suggesting that diameter of the fibers affects neither phagocytosis nor intracellular localization. It is known that phagocytosed particles can be exocytosed via non-vesicle related secretion or lysosome secretion [30]. The short fibers that were phagocytosed may also be exocytosed and localized on the surface, thus making the difference in their intracellular localization. However, further research is required to explain why long fibers tend to remain inside cells.

### Survival of cells after asbestos phagocytosis

**Fig. 5:**
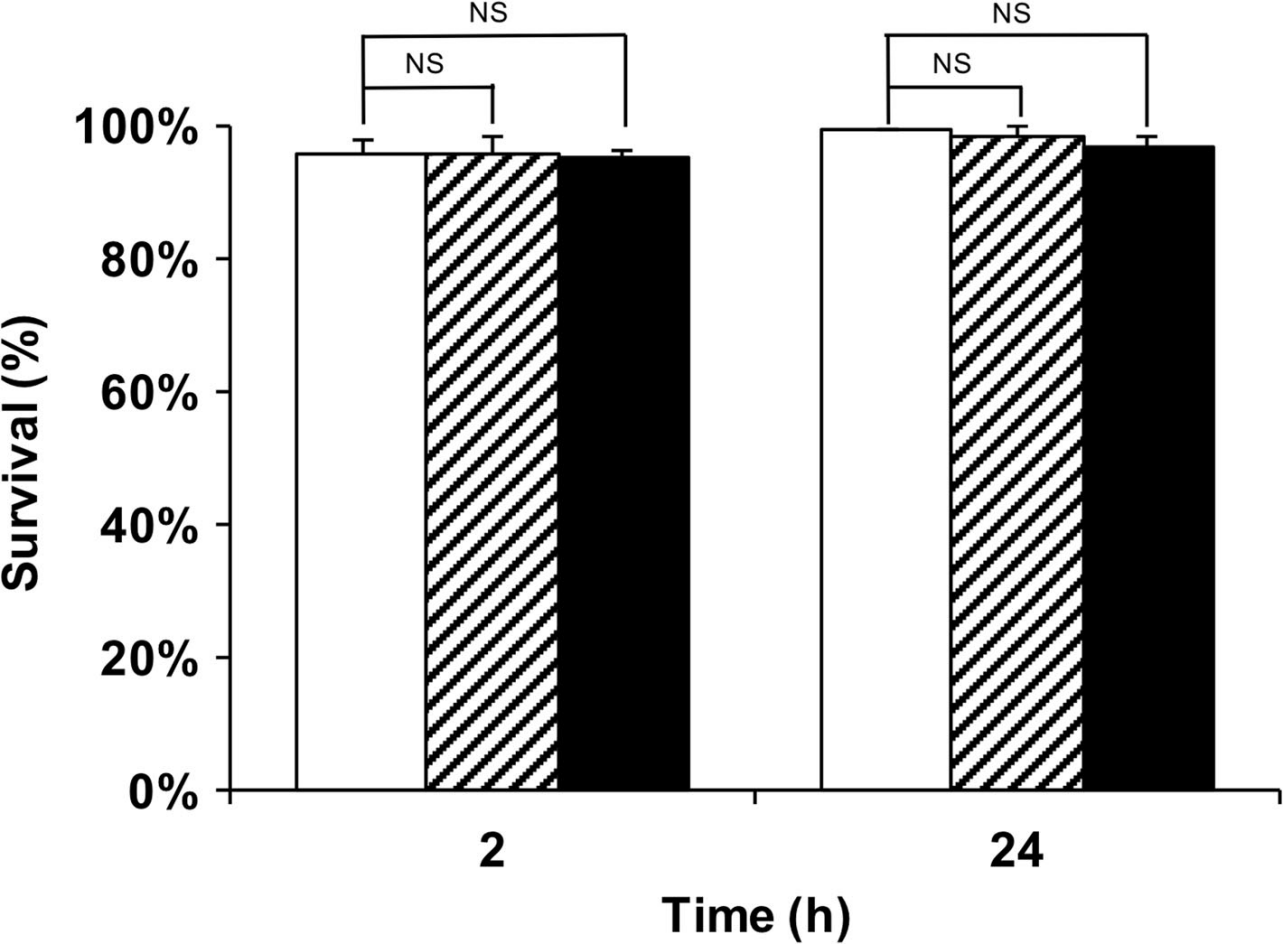
Survival of RAW 264.7 cells during asbestos phagocytosis. Cells were incubated in the absence of asbestos (white bars) or in the presence of 25 μg/mL of either unlabeled (striped bars) or fluorescently labeled (black bars) asbestos for 2 h or 24 h. The data represent the mean of three experiments with at least 100 cells per sample, and the error bars represent the standard deviation. Statistical analysis was performed using the two-tailed unpaired Student’s *t*-test. NS, not significant

**Fig. 6:**
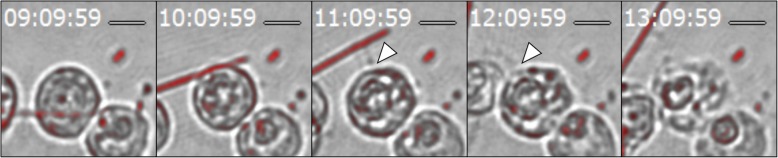
Time-lapse analysis of immediate cell death. Arrowheads indicate the site of membrane rapture. Time (h: min: sec) is shown in the upper left corner. Scale bar = 10 μm

Reconstructed CLSM images revealed that 58.8% of the cells (40/68) had phagocytosed asbestos fibers by the 2-h time-point. Of these, 16.2% (11/68) phagocytosed asbestos fibers longer than 10 μm. To investigate whether asbestos phagocytosis led to immediate cell death, cell survival was measured at 2 h and at 24 h via propidium iodide uptake. Over 96% of cells remained alive 24 h after the addition of both unlabeled and fluorescently labeled asbestos fibers (Fig. 5), suggesting that asbestos phagocytosis does not lead to immediate cell death. It has been suggested that frustrated phagocytosis results in failure of membrane closure, and consequent leakage of cell contents [14]. Such leakage could lead to immediate cell death. In the present study the time-course of the leakage of the cell contents of 153 cells that phagocytosed asbestos fibers was observed. Leakage of cell contents and subsequent cell death was only observed when an asbestos fiber was physically pulled from a cell by an external force (Fig. 6). In the case depicted in Fig. 6, each end of a single long fiber was phagocytosed by a different macrophage. Each macrophage internalized “their end” for approximately 10 h, before the long fiber was apparently pulled from one of the macrophages via physical forces exerted by the other macrophage. It was only then that a large amount of cellular content leaked due to the ruptured membrane, and the cell rapidly died. Only two such cell deaths were observed in 153 cells, suggesting that this may not be a major initial event related to the asbestos-associated cell toxicity, at least not in vitro.

### Macrophage division after asbestos phagocytosis

In in vitro asbestos-binding experiments using a protein solution extracted from cultured human lung cells, MacCorkle et al. [31] reported that several cytoskeletal proteins including ß-tubulin, vimentin, lamin A/C, and actin exhibited the capacity to bind to asbestos. These proteins are required for mitosis and related cytoskeletal functions. Actin and several heat-shock proteins contained in a macrophage protein extract were also identified as asbestos-binding proteins in the present study (Additional file 2), and additionally, internalized asbestos fibers were partly co-localized with actin (Fig. 3c).

**Fig. 7:**
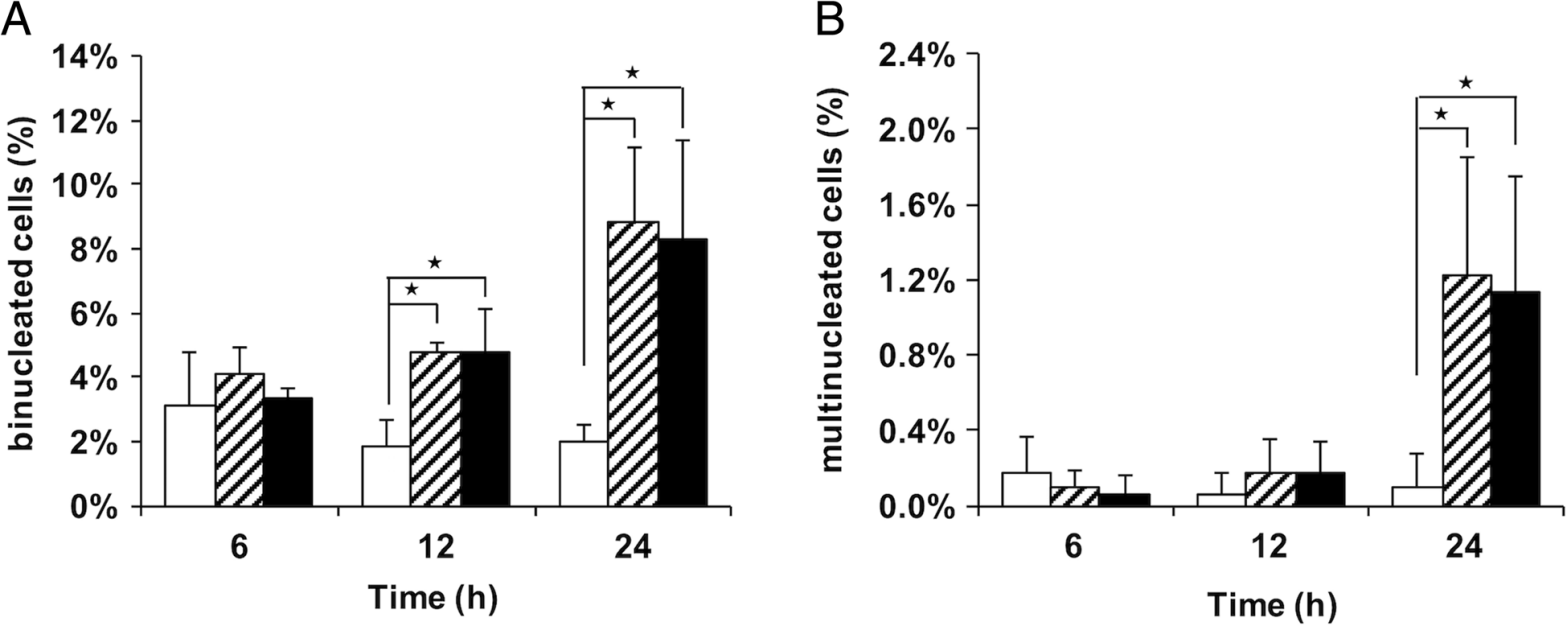
Asbestos-induced binucleation and multinucleation in RAW 264.7 cells. Graphs show the frequencies of binucleated (**a**) and multinucleated cells (**b**). Cells were incubated in the absence of asbestos (white bars) or in the presence of 25 μg/mL of either unlabeled (striped bars) or fluorescently labeled (black bars) asbestos for 6 h, 12 h, or 24 h. The data represent the mean of three experiments with at least 500 cells per sample, and the error bars represent the standard deviation. Statistical analysis was performed using the two-tailed unpaired Student’s *t*-test. ^★^*p* < 0.05 vs. absence of asbestos

The cytoskeletal protein-binding property of asbestos may affect the dynamic process of cell division. In the current study the generation of binucleated cells in vitro in the presence and absence of asbestos fibers in the culture medium was quantified over a time-course. The number of binucleated cells was approximately four times greater 24 h after the addition of asbestos (Fig. 7a). The frequency of cells with more than three nuclei was approximately 10-fold higher than it was cultures that were not exposed to asbestos (Fig. 7b). In cultures exposed to asbestos, neither the frequencies of formation of binucleated cells, nor the frequencies of cells with more than three nuclei differed significantly depending on whether the asbestos fibers were fluorescently labeled (Fig. 7a and b).

**Fig. 8:**
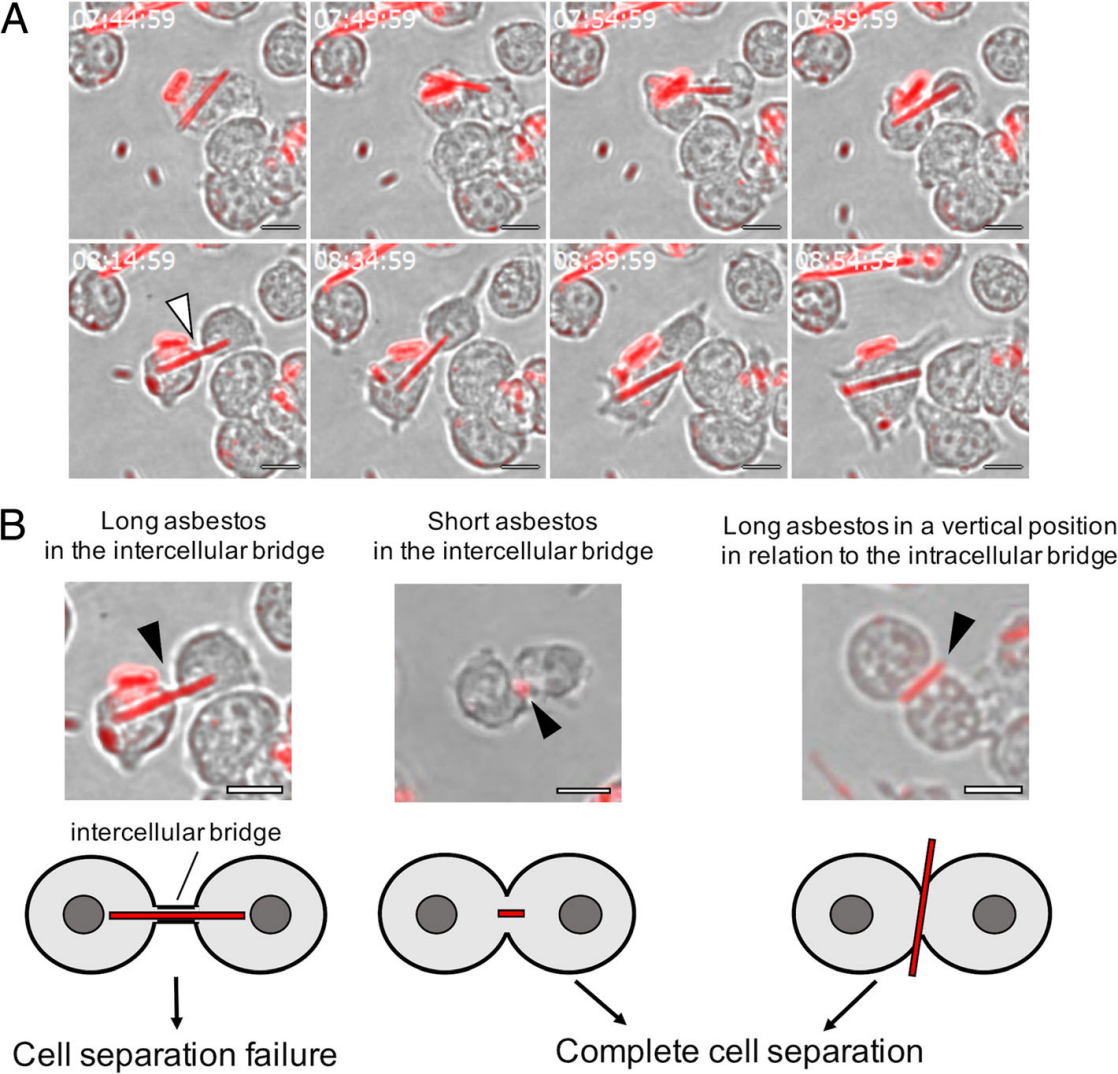
Live-cell imaging of asbestos-induced binucleation. **a** Formation of a binucleated cell in the presence of asbestos fibers. The images are from the field of view shown in Additional file 3. Time (h: min: sec) is shown in the upper left corner. **b** Diagrammatic representations of asbestos-induced binucleation. Scale bar = 10 μm

The formation of binucleated cells can evidently result from a situation, in which a long internalized fiber is shared by two daughter cells during cell division (Fig. 8a, arrowhead at 08:14:59). At the last stage of cell division the resulting two daughter cells should be separated. However, in the case depicted in Fig. 8a a long asbestos fiber shared by two daughter cells intercellularly seemed to retard complete separation (Fig. 8a at 08:34:59). One daughter cell would likely have died if the other had exerted sufficient force to pull the long fiber from the other’s cytoplasm such that its membrane was terminally ruptured, as in the scenario depicted in Fig. 6. Instead however, in this instance the two daughter cells fused and the resulting single cell survived (Fig. 8a at 08:39:59). The binding affinity of asbestos to actin may block the removal of asbestos fibers from cells and retard cell separation. The previously reported MGC formation observed in asbestos-exposed mouse lungs [18] may be attributable to the prevention of complete cell separation by internalized fibers, which is probably independent of IL-4, which induces fusion [20, 21].

Jensen et al. [16] and Cortez and Machado-Santelli [32] proposed a similar mechanism for the formation of binucleated cells in the presence of asbestos. To further investigate binucleated cell formation we monitored 53 individual cell divisions of cells that had phagocytosed asbestos (9 long fibers and 44 short fibers), and of these, 4 divisions of cells that had phagocytosed long fibers did not ultimately result in complete cell separation. Interestingly, in these cases the two daughter cells resulting from initial division fused thereafter, resulting in the formation of a binucleated cell. A typical pattern of asbestos-facilitated binucleated cell formation is shown in Fig. 8a, and an additional movie file depicts the process in more detail (Additional file 3). The mean length of the internalized asbestos fibers in the four cells that failed to complete cell separation was 13.9 ± 1.9 μm (mean ± SE), suggesting that short fibers may not inhibit cell separation. Notably in this regard, in the 21 instances observed the presence of a short fiber in the intercellular bridge did not appear to affect cell separation (Fig. 8b, middle panel). Furthermore, even the presence of a long asbestos fiber in a vertical position in relation to the intracellular bridge (4 cells) did not inhibit cell separation (Fig. 8b, right panel), suggesting that it may primarily be the presence of a long asbestos fiber aligned with the intercellular bridge that inhibits cell separation.

### Chromosomal instability and aneuploidy in binucleated cells

Asbestos is an established carcinogen that causes such as pleural mesothelioma and lung cancers [3–6]. Here we showed that frustrated phagocytosis itself does not lead to immediate cell death, but blocks cell separation, resulting in the formation of a binucleated cell. Asbestos-induced chromosomal instability and consequent aneuploidy, a hallmark of cancer cells, has been observed in various types of in vitro cultured mammalian cells [32–39]. Recently, Zhang et al. [40] has demonstrated that aneuploid cells are generated from binucleated cells through multipolar mitosis or bipolar mitosis with chromosome nondisjunction. Changes in the copy numbers of chromosomes or large chromosomal regions in aneuploid cells significantly alter the expression of several hundreds of genes. In cancer, aneuploidy can be an effective mechanism for generating phenotypic variation that correlates with poor prognosis, increased metastatic potential and drug resistance, thus suggesting that aneuploidy generated from asbestos-induced binucleated cells likely provides an adaptive advantage of cancerous cells over diploid cells in a healthy tissue [17, 41, 42].

### Future application of fluorescently labeled inorganic binding proteins/peptides

Our first trial using fluorescently labeled asbestos enabled us to visualize the dynamic biological processes that accompany the cellular internalization of asbestos fibers. Other types of particles and nanoparticles may adversely affect the environment and human health [43, 44]. Some researchers have suggested that the release of nanoparticles into the environment constitutes a new type of pollution [43, 44]. FM of live cells with fluorescent tags provides a range of tools to investigate many cellular processes via microscopy. The sensitivity of FM is sufficient for the detection of single chrysotile fibrils (30–35 nm in diameter) which are not visible under phase contrast microscopy [25], indicating that the method can be applied in the field of live-cell imaging involving nanoparticles. Peptides that bind to harmful or potentially harmful nanomaterials such as carbon nanotubes, silver, platinum, aluminum oxide, silica, titanium dioxide, zinc oxide, fullerenes, and polystyrene have been reported [45]. Although the resolution of conventional FM is limited by optical diffraction, the method will likely lead to novel applications in nanotoxicological studies.

## Conclusions

Our method using fluorescently labeled asbestos enabled us to visualize internalized both long and short asbestos fibers. Visualization of the dynamic biological processes that occur during and after the internalization of asbestos fibers indicated that (i) frustrated phagocytosis itself does not lead to immediate cell death unless the asbestos fiber is physically pulled from the cell by an external force, and (ii) macrophages that have phagocytosed asbestos can divide but sometimes the resulting daughter cells fuse, leading to the formation of a binucleated cell. This fusion only seemed to occur when a comparatively long asbestos fiber (> 10 μm) was localized in the proximity of and in parallel with the intercellular bridge between two daughter cells. This methodology can be applied in the field of live-cell imaging involving nanoparticles and contribute to nanotoxicological studies.

## Additional files

Additional file 1: Phagocytosis of asbestos fibers by RAW 264.7 cells. RAW 264.7 cells were monitored after adding fluorescently labeled asbestos (red). Images were then obtained continuously every 5 min. The movie is formatted at three frames per second. Time (h: min: sec) is shown in the upper left corner. Scale bar =10 μm. (MP4 445 kb)

Additional file 2: **Figure S1.** Asbestos-binding proteins in RAW 264.7 cells. RAW 264.7 cell lysate was incubated with asbestos. Proteins bound to asbestos were subsequently separated via 12.5% SDS-polyacrylamide gel electrophoresis. Protein bands were excised from the gel, digested with sequencing-grade trypsin, and analyzed via liquid chromatography-tandem mass spectrometry. The peptide fingerprints obtained were used in protein searches conducted using Mascot. The proteins identified are shown on the right. (DOCX 205 kb)

Additional file 3: Formation of a binucleated cell in the presence of asbestos fibers. RAW 264.7 cells were monitored after adding fluorescently labeled asbestos (red). Images were then obtained continuously every 5 min. The movie is formatted at three frames per second. Time h: min: sec) is shown in the upper left corner. Scale bar =10 μm. (MP4 312 kb)

## References

[1] Mossman B, Bignon J, Corn M, Seaton A, Gee J (1990). Asbestos – scientific developments and implications for public-policy. Science..

[2] Perkins RL, Harvey BW. Test method: method for the determination of asbestos in bulk building materials, EPA/600/R-93/116. 1993. https://www.nist.gov/sites/default/files/documents/nvlap/EPA-600-R-93-116.pdf. Accessed 21 Jan 2019.

[3] Kanarek MS (2011). Mesothelioma from chrysotile asbestos: update. Ann Epidemiol.

[4] Davis JM, Beckett ST, Bolton RE, Collings P, Middleton AP (1978). Mass and number of fibres in the pathogenesis of asbestos-related lung disease in rats. Br J Cancer.

[5] Theakston F, Theakston F (2000). Asbestos. Air quality guidelines for Europe. 2nd ed. World Health Organization, regional Office for Europe.

[6] Ramos-Nino ME, Testa JR, Altomare DA, Pass HI, Carbone M, Bocchetta M, Mossman BT (2006). Cellular and molecular parameters of mesothelioma. J Cell Biochem.

[7] Furuya S, Takahashi K (2017). Experience of Japan in achieving a Total ban on Asbestos. Int J Environ Res Public Health.

[8] Mossman B, Churg A (1998). Mechanisms in the pathogenesis of asbestosis and silicosis. Am J Respir Crit Care Med.

[9] Kamp DW, Weitzman SA (1999). The molecular basis of asbestos induced lung injury. Thorax..

[10] Lehnert B (1993). Defense mechanisms against inhaled particles and associated particle-cell interactions. Rev Mineral.

[11] Oberdorster G (1994). Macrophage-associated responses to chrysotile. Ann Occup Hyg.

[12] Schinwald A, Murphy FA, Prina-Mello A, Poland CA, Byrne F, Movia D, Glass JR, Dickerson JC, Schultz DA, Jeffree CE (2012). The threshold length for fiber-induced acute pleural inflammation: shedding light on the early events in asbestos-induced mesothelioma. Toxicol Sci.

[13] Dörger M, Münzing S, Allmeling AM, Messmer K, Krombach F (2001). Differential responses of rat alveolar and peritoneal macrophages to man-made vitreous fibers in vitro. Environ Res.

[14] Schinwald A, Donaldson K (2012). Use of back-scatter electron signals to visualise cell/nanowires interactions in vitro and in vivo; frustrated phagocytosis of long fibres in macrophages and compartmentalisation in mesothelial cells in vivo. Part Fibre Toxicol.

[15] Dostert C, Petrilli V, Van Bruggen R, Steele C, Mossman B, Tschopp J (2008). Innate immune activation through Nalp3 inflammasome sensing of asbestos and silica. Science..

[16] Jensen CG, Jensen LC, Rieder CL, Cole RW, Ault JG (1996). Long crocidolite asbestos fibers cause polyploidy by sterically blocking cytokinesis. Carcinogenesis..

[17] Barrett JC, Tsutsui T, Tsly T, Harris CC, Liotta LA (1990). Oshimura M. Genetic mechanisms in carcinogenesis and tumor progression.

[18] Prieditis H, Adamson IY (1996). Alveolar macrophage kinetics and multinucleated giant cell formation after lung injury. J Leukoc Biol.

[19] Helming L, Gordon S (2009). Molecular mediators of macrophage fusion. Trends Cell Biol.

[20] Kao WJ, McNally AK, Hiltner A, Anderson JM (1995). Role for interleukin-4 in foreign-body giant cell formation on a poly(etherurethane urea) in vivo. J Biomed Mater Res.

[21] Prokop S, Heppner FL, Goebel HH, Stenzel W (2011). M2 polarized macrophages and giant cells contribute to myofibrosis in neuromuscular sarcoidosis. Am J Pathol.

[22] Blake DJ, Bolin CM, Cox DP, Cardozo-Pelaez F, Pfau JC (2007). Internalization of Libby amphibole asbestos and induction of oxidative stress in murine macrophages. Toxicol Sci.

[23] Wang NS, Jaurand MC, Magne L, Kheuang L, Pinchon MC, Bignon J (1987). The interactions between asbestos fibers and metaphase chromosomes of rat pleural mesothelial cells in culture. A scanning and transmission electron microscopic study. Am J Pathol.

[24] Gianoncelli A, Kourousias G, Cammisuli F, Cassese D, Rizzardi C, Radillo O, Lazzarino M, Pascolo L (2017). Combined use of AFM and soft X-ray microscopy to reveal fibres' internalization in mesothelial cells. Analyst..

[25] Ishida T, Alexandrov M, Nishimura T, Minakawa K, Hirota R, Sekiguchi K, Kohyama N, Kuroda A (2012). Evaluation of sensitivity of fluorescence-based Asbestos detection by correlative microscopy. J Fluoresc.

[26] Ishida T, Alexandrov M, Nishimura T, Minakawa K, Hirota R, Sekiguchi K, Kohyama N, Kuroda A (2010). Selective detection of airborne Asbestos fibers using protein-based fluorescent probes. Environ Sci Technol.

[27] Iloki Assanga SB, Gil-Salido AA, Lewis Luján LM, Rosas-Durazo A, Acosta-Silva AL, Rivera-Castañeda EG, Rubio-Pino JL (2013). Cell growth curves for different cell lines and their relationship with biological activities. Int J Biotechnol Mol Biol Res.

[28] Ishihara Y (2001). In vitro studies on biological effects of fibrous minerals. Ind Health.

[29] Funahashi S, Okazaki Y, Ito D, Asakawa A, Nagai H, Tajima M, Toyokuni S (2015). Asbestos and multi-walled carbon nanotubes generate distinct oxidative responses in inflammatory cells. J Clin Biochem Nutr.

[30] Oh N, Park JH (2014). Endocytosis and exocytosis of nanoparticles in mammalian cells. Int J Nanomedicine.

[31] MacCorkle RA, Slattery SD, Nash DR, Brinkley BR (2006). Intracellular protein binding to asbestos induces aneuploidy in human lung fibroblasts. Cell Motil Cytoskeleton.

[32] Cortez BA, Machado-Santelli GM (2008). Chrysotile effects on human lung cell carcinoma in culture: 3-D reconstruction and DNA quantification by image analysis. BMC Cancer.

[33] Ruosaari ST, Nymark PE, Aavikko MM, Kettunen E, Knuutila S, Hollmen J, Norppa H, Anttila SL (2008). Aberrations of chromosome 19 in asbestos-associated lung cancer and in asbestos-induced micronuclei of bronchial epithelial cells in vitro. Carcinogenesis..

[34] Cortez Bde A, Quassollo G, Caceres A, Machado-Santelli GM (2011). The fate of chrysotile-induced multipolar mitosis and aneuploid population in cultured lung cancer cells. PLoS One.

[35] Hesterberg TW, Barrett JC (1985). Induction by asbestos fibers of anaphase abnormalities: mechanism for aneuploidy induction and possibly carcinogenesis. Carcinogenesis..

[36] Funaki K, Everitt J, Bermudez E, Walker C (1991). Trisomy of rat chromosome 1 associated with mesothelial cell transformation. Cancer Res.

[37] Cleaver AL, Bhamidipaty K, Wylie B, Connor T, Robinson C, Robinson BW, Mutsaers SE, Lake RA (2014). Long-term exposure of mesothelial cells to SV40 and asbestos leads to malignant transformation and chemotherapy resistance. Carcinogenesis..

[38] Qi F, Okimoto G, Jube S, Napolitano A, Pass HI, Laczko R, Demay RM, Khan G, Tiirikainen M, Rinaudo C (2013). Continuous exposure to chrysotile asbestos can cause transformation of human mesothelial cells via HMGB1 and TNF-alpha signaling. Am J Pathol.

[39] Lechner JF, Tokiwa T, LaVeck M, Benedict WF, Banks-Schlegel S, Yeager H, Banerjee A, Harris CC (1985). Asbestos-associated chromosomal changes in human mesothelial cells. Proc Natl Acad Sci U S A.

[40] Zhang T, Lv L, Huang Y, Ren X, Shi Q (2017). Chromosome nondisjunction during bipolar mitoses of binucleated intermediates promote aneuploidy formation along with multipolar mitoses rather than chromosome loss in micronuclei induced by asbestos. Oncotarget..

[41] Gordon DJ, Resio B, Pellman D (2012). Causes and consequences of aneuploidy in cancer. Nat Rev Genet.

[42] Durrbaum M, Storchova Z (2016). Effects of aneuploidy on gene expression: implications for cancer. FEBS J.

[43] Magrez A, Kasas S, Salicio V, Pasquier N, Seo JW, Celio M, Catsicas S, Schwaller B, Forro L (2006). Cellular toxicity of carbon-based nanomaterials. Nano Lett.

[44] Poland CA, Duffin R, Kinloch I, Maynard A, Wallace WA, Seaton A, Stone V, Brown S, Macnee W, Donaldson K (2008). Carbon nanotubes introduced into the abdominal cavity of mice show asbestos-like pathogenicity in a pilot study. Nat Nanotechnol.

[45] Kuroda A, Alexandrov M, Nishimura T, Ishida T (2016). Rapid on-site detection of airborne asbestos fibers and potentially hazardous nanomaterials using fluorescence microscopy-based biosensing. Biotechnol J.

