# Supplementary material for: Live-cell imaging of macrophage phagocytosis of asbestos fibers under fluorescence microscopy
Source: Genes Environ. 2019 Jun 5;41:14. doi: 10.1186/s41021-019-0129-4 (PMC6549298; doi:10.1186/s41021-019-0129-4)
Supplement: Supplementary file 2 — Figure S1. Asbestos-binding proteins in RAW 264.7 cells. RAW 264.7 cell lysate was incubated with asbestos. Proteins bound to asbestos were subsequently separated via 12.5% SDS-polyacrylamide gel electrophoresis. Protein bands were excised from the gel, digested with sequencing-grade trypsin, and analyzed via liquid chromatography-tandem mass spectrometry. The peptide fingerprints obtained were used in protein searches conducted using Mascot. The proteins identified are shown on the right. (DOCX 205 kb) [file 41021_2019_129_MOESM2_ESM.docx]

**Additional file 2**

**Asbestos binds a selective protein profile in RAW 264.7 cells.**

RAW 264.7 cells were suspended in PBS and disrupted via ultrasonication (Branson, CT, USA). The lysate was centrifuged at 10,000 *g* for 10 min. The protein concentration of the cleared supernatant was adjusted to 0.5 mg/mL with PBS. Asbestos (0.2 mg) was added to 0.5 mL of the diluted supernatant, incubated for 30 min at room temperature with vortex mixing, and precipitated via centrifugation at 12,000 *g* for 3 min. The precipitated fibers were washed three times with 0.5 mL of PBS. Proteins bound to fibers were eluted by boiling for 5 min in 500 μL of SDS-sample buffer containing 100 mM Tris-HCl (pH 6.8), 2% SDS, 8% glycerol, 4% 2-mercaptoethanol, and 0.05% bromophenol blue, then separated via 12.5% SDS-polyacrylamide gel electrophoresis. Protein bands were excised from the gel, digested with sequencing-grade trypsin (Promega, Madison, WI, USA), and analyzed using liquid chromatography-tandem mass spectrometry (LTQ Orbitrap XL, Thermo Fisher Scientific, Hanover Park IL, USA). The peptide fingerprints obtained via LTQ Orbitrap XL were used in protein searches conducted using Mascot (Matrix science Ltd., London, UK).


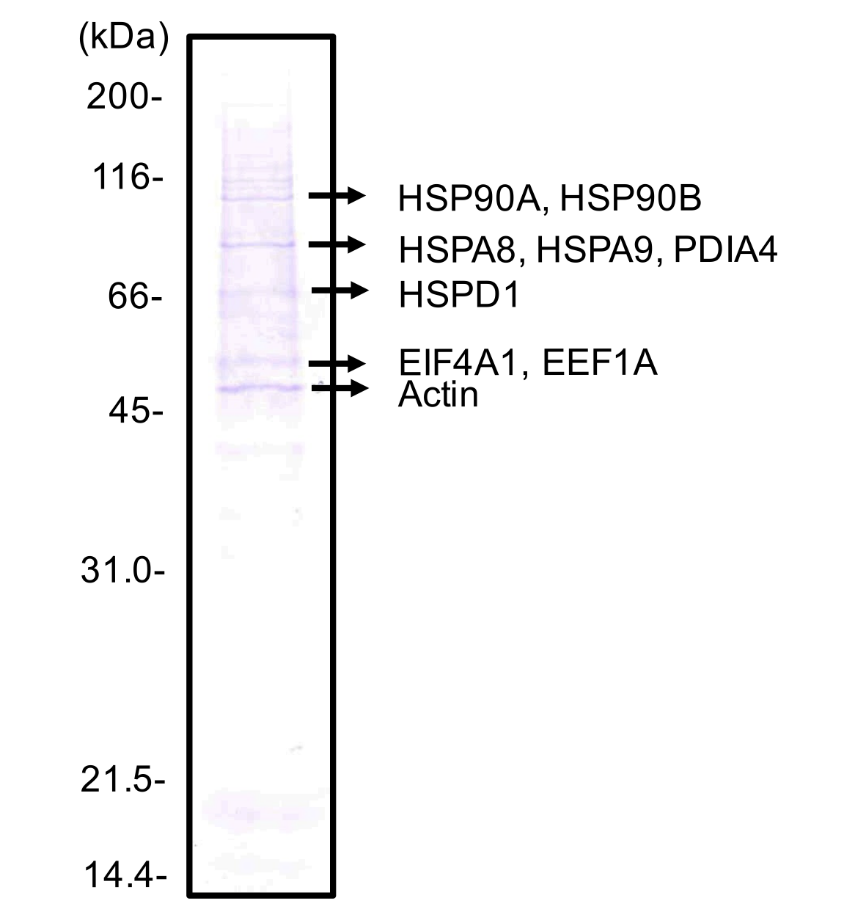


**Fig. S1.** Asbestos-binding proteins in RAW 264.7 cells. RAW 264.7 cell lysate was incubated with asbestos. Proteins bound to asbestos were then separated via 12.5% SDS-polyacrylamide gel electrophoresis. Protein bands were excised from the gel, digested with sequencing-grade trypsin, and analyzed using liquid chromatography-tandem mass spectrometry. The peptide fingerprints obtained were used in protein searches conducted via Mascot. The proteins identified are shown on the right.
